# Lossless Data Compression for Time-Series Sensor Data Based on Dynamic Bit Packing

**DOI:** 10.3390/s23208575

**Published:** 2023-10-19

**Authors:** Sang-Ho Hwang, Kyung-Min Kim, Sungho Kim, Jong Wook Kwak

**Affiliations:** 1 Gyeongbuk Institute of IT Convergence Industry Technology, Gyeongsan 38463, Republic of Korea; shhwang@gitc.or.kr (S.-H.H.); shk@gitc.or.kr (S.K.); 2 Department of Computer Engineering, Yeungnam University, Gyeongsan 38541, Republic of Korea; bl43a@ynu.ac.kr

**Keywords:** sensor data, lossless compression, time series data, bit packing, bit depth level

## Abstract

In this paper, we propose a bit depth compression (BDC) technique, which performs bit packing by dynamically determining the pack size based on the pattern of the bit depth level of the sensor data, thereby maximally reducing the space wastage that may occur during the bit packing process. The proposed technique can dynamically perform bit packing according to the data’s characteristics, which may have many outliers or several multidimensional variations, and therefore has a high compression ratio. Furthermore, the proposed method is a lossless compression technique, which is especially useful as training data in the field of artificial intelligence or in the predictive analysis of data science. The proposed method effectively addresses the spatial inefficiency caused by unpredictable outliers during time-series data compression. Additionally, it offers high compression efficiency, allowing for storage space savings and optimizing network bandwidth utilization while transmitting large volumes of data. In the experiment, the BDC method demonstrated an improvement in the compression ratio of up to 247%, with 30% on average, compared with other compression algorithms. In terms of energy consumption, the proposed BDC also improves data transmission using Bluetooth up to 34%, with 18% on average, compared with other compression algorithms.

## 1. Introduction

**Figure 1:**
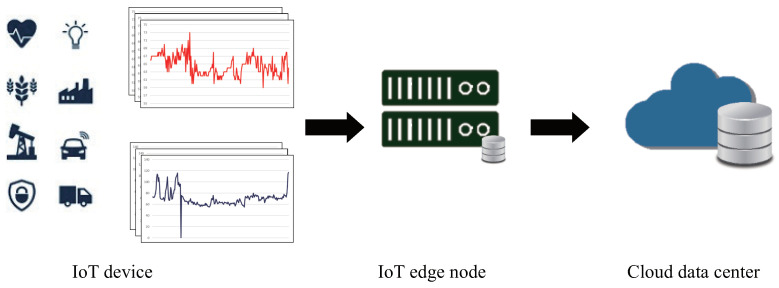
Network topology from IoT sensor device to cloud data center.

Sensors are used in various fields, such as in autonomous vehicles, wearable devices, smart farms, and smart factories, for collecting sensing data in real time [1,2,3,4]. These sensors collect time-series data that are correlated with each other. Then, the data collected from the various sensors are transmitted to edge devices or cloud data centers, as shown in Figure 1. With the introduction of digital twins and smart factories, more than 20 billion of IoT sensors were in use in environments such as factories, buildings, and cities in 2020 [5]. According to a recent study, the number of connected IoT devices is expected to reach 30 billion in 2030 [6].

Connecting to the virtual environment will require the use of multiple sensors in the real world, and the amount of data collected by these sensors will cause considerable communication load when transmitting to a data server, such as cloud data centers. According to Intel Corporation, for example, cameras and light detection and ranging (LiDAR) sensors currently installed in autonomous vehicles generate 4 TB of data every 90 min. To transmit and manage the enormous amount of time-series data generated by these sensors, many studies on time-series data compression have recently been conducted [7,8,9,10,11,12,13,14,15,16,17,18,19,20,21,22,23,24,25,26,27,28,29,30,31,32,33,34,35].

Generally, sensors collect data including various types of noise, which is caused by their inherent inaccuracies or environmental factors. To address this situation, lossy compression techniques are frequently utilized to control noise and maximize the compression ratio within an allowable range of data similarity. In general, lossy compression algorithms have a higher compression ratio than lossless ones. Although lossy compression algorithms also have a significant advantage in data transmission owing to their high compression ratios, they can lead to a loss of important information, such as the subtle differences used to distinguish data classes. To address this issue, several studies on lossless techniques have been conducted [18,19,20,22]. However, the existing lossless algorithms do not accurately reflect data characteristics when the data have have many outliers or several multidimensional variations (i.e., data robustness); hence, their compression efficiency is not high enough and not suitable for compressing big data.

To resolve this problem and increase compression efficiency, this study proposes a bit depth compression (BDC) technique. The BDC algorithm performs bit packing by dynamically determining the size of the bit pack based on the pattern of the bit depth level of the sensor data, thereby reducing the amount of space that may be wasted during its bit packing. Furthermore, the proposed technique is a lossless compression that can be used to compress time-series data that have many outliers or several multidimensional variations. Therefore, the proposed technique can more efficiently perform dynamic bit packing based on the data robustness, resulting in a high compression ratio. Our contributions are summarized as follows:

- We propose a time-series data compression technique based on bit packing by dynamically determining the size of the bit pack based on the pattern of the bit depth level of the sensor data.
- Our proposal effectively addresses the spatial inefficiency caused by unpredictable outliers during time-series data compression, and BDC offers high compression efficiency, allowing for storage space savings and optimizing network bandwidth utilization while transmitting large volumes of data.

The remainder of this paper is organized as follows: Section 2 presents the background knowledge and related literature. Section 3 describes the BDC technique that performs lossless compression through dynamic bit packing according to the bit depth level. Section 4 evaluates the performance of the BDC technique against existing compression techniques. Finally, Section 5 concludes the paper.

## 2. Background and Related Literature

Data compression has two types: lossy compression, which results in information loss during encoding; and lossless compression, which does not result in information loss [8]. Lossy compression techniques have been extensively studied because of the benefits associated with their high compression ratio [9,10,11,12,13,14]. The compression of these algorithms is based on generated patterns or mathematical models such as temporal sampling or regularity based on specific data features. Generally, lossy compression techniques are suitable for applications where the loss of data is acceptable.

Lossless compression methods are more suitable for applications that require the fine control of sensor data [15,16,17,18,19,20,21,22]. Lossless compression techniques include well-known coding schemes, such as Huffman coding or run-length encoding; however, these coding schemes are inefficient for time-series data compression [16,17]. Instead, in the field of IoT sensor data, the forecasting algorithm is widely used for the compression of time-series data, which is characterized as a data sequence with time intervals [17]. The time-series forecasting algorithm predicts the next value by creating a mathematical model from the given time-series data. These algorithms include techniques such as delta coding, delta of deltas coding, or auto-regressive (AR) coding [19,20,21]. Delta coding or delta of deltas coding stores the differences between consecutive numbers instead of raw values from an array of numbers with the initial values. In general, these delta coding schemes show a high compression ratio for time-series data with small variations, but the compression efficiency is seriously degraded for data with several multidimensional variations.

Recent works have focused on utilizing some models such as AR and recurrent neural network (RNN) to improve the forecasting accuracy of time-series data with forecasting algorithms. These models can help achieve a relatively high compression ratio compared with delta coding schemes due to the high prediction accuracy of time-series data, but they are difficult to utilize for terminal end devices that are sensitive to energy consumption due to their increased computational complexity [22].

Among the lossless compression techniques used for time-series data, the GORILLA algorithm was designed for the time-series database of Facebook [19]. This GORILLA algorithm reduces the number of bits of timestamp values included in time-series data as delta of deltas and reduces the data bits by using XOR encoding. This technique exhibits high compression efficiency for time-series data where the values change slowly and are consistently similar but has a disadvantage in that compression efficiency drops sharply for time-series data that change rapidly or have no patterns.

Idrees et al. proposed the lossless electroencephalography (EEG) data compression (LEDaC) technique based on the Internet of medical things (IoMT) for fog computing networks [24]. This technique combines DBSCAN clustering with Huffman encoding. DBSCAN is used to group EEG data and apply Huffman encoding to each group, compressing the time-series data. This technique has the advantage of achieving high compression rates without loss of EEG data, but it may be challenging to apply it to other types of time-series data with non-regular patterns.

The Sprintz algorithm was proposed by Davis Blalock et al. as a method for compressing integer time series data [22]. The Sprintz algorithm includes a forecasting algorithm that predicts time-series data, a bit packing process that reduces bits for an array of the error between predicted values and actual values, a run-length encoding that reduces repeated values in which runs of 0, and Huffman coding that is applied as entropy coding. This technology reduces data volume through forecasting encoding and then improves the compression ratio by sequentially applying existing lossless compression algorithms. However, since the Sprintz algorithm statically samples sensor data at a fixed size, the compression ratio varies greatly depending on data type and pattern.

## 3. Data Compression Technique Based on Dynamic Bit Packing Using Bit Depth

In this section, we propose a bit depth compression (BDC) algorithm that dynamically performs bit packing for time-series data compression. We first show the structure of the BDC and then explain how the BDC works with example scenarios.

### 3.1. Structure of BDC

**Figure 2:**
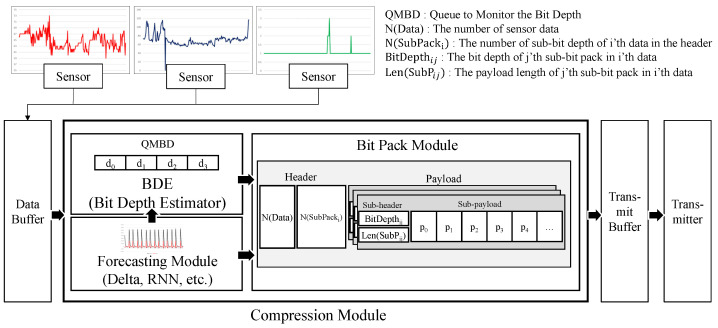
Structure of the proposed BDC algorithm.

The proposed BDC is a bit packing algorithm that dynamically performs compression according to the bit depth of a value extracted from a forecasting algorithm such as delta or RNN. Figure 2 shows the overall structure of the proposed BDC method.

As shown in Figure 2, the data collected from sensors are stored in a data buffer and compressed before being transmitted through a transmitter. First, the forecasting module (FM), which performs compression by predicting the future values of time-series data, can utilize various forecasting techniques, such as delta, delta of deltas, and RNN encoding. Because the values predicted through the FM have various levels of bit depth, a lot of space in the payload may be filled with zeros. The proposed technique varies the payload size to reduce space wastage. To this end, our technique utilizes the bit depth level of the data collected through a bit depth estimator (BDE) when performing bit packing. The BDE includes a queue to monitor the bit depth (QMBD) that traces the bit depth level of the error value generated by FM, and this monitoring result is used by the bit packing module in the next stage. The bit pack module performs bit packing by splitting it into several sub-packs according to the bit depth information. The bit-packed values are then stored in a transmit buffer and transmitted by a transmitter according to the transmission policy. The proposed technique employs the dynamic partitioning of sensor data based on the bit depth of the values during compression, instead of statically packing them into a fixed size. Therefore, this BDE offers the advantage of a high compression ratio.

### 3.2. Procedure of the BDC

We explain the procedure and algorithm of BDC with example scenarios. To explain the operations and contributions of the BDC algorithm, we first show the operation of the previous algorithm.

**Figure 3:**
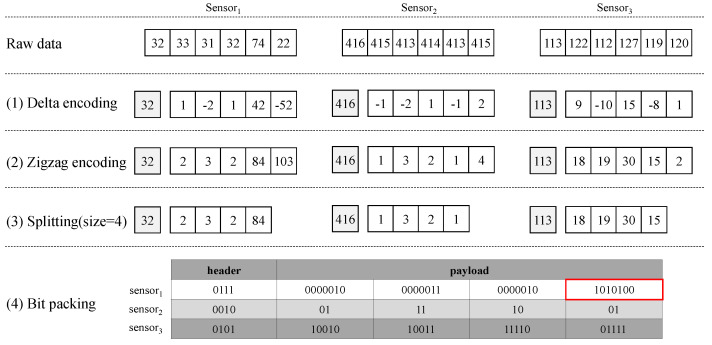
Example of compression using delta encoding. A bit depth level for each sensor datum is stored in the header, and a zigzag-encoded value is stored in the payload.

Figure 3 shows the values extracted after delta-based forecasting of time-series data in the Sprintz algorithm for discussion to improve the previous technique. In order to demonstrate the potential inefficiencies in the existing algorithms for time-series data compression, Figure 3 illustrates the compression process for three distinct sensor data streams. Figure 3 presents multiple streams for illustrative purposes, showing our proposed compression has no correlation of multiple data streams and focuses on single data streams. Figure 3 depicts the following steps: (1) Time-series data collected from each sensor are compressed via delta encoding, which predicts the next value of the time-series data and calculates the difference from the actual value. In contrast to XOR encoding, where the initial value significantly influences compression performance, delta forecasting encoding only considers the difference between consecutive sensor data, making the initial value relatively less important. We use the initial value detected in the first stage, and the value is transmitted separately to the server to ensure the accurate reconstruction of subsequent data points. (2) This difference in value may be a negative number. To handle this, the zigzag algorithm converts the sign bit from the most significant bit (MSB) to the least significant bit (LSB). In the zigzag algorithm, the encoding for a given input value *n*, with fixed *k*-bit integers, is defined as $\left(n\gg \left(k-1\right)\right)\wedge \left(n\ll 1\right)$. Similarly, the decoding process can be expressed as $\left(n\gg 1\right)\wedge -\left(n\&1\right)$, where ∧ represents XOR, & represents AND, ≪ denotes a bitwise left shift, and ≫ denotes a bitwise right shift. (3) The Sprintz performs bit packing of the data into blocks of samples through splitting to respond to rapid entropy changes or to apply different encodings for each sensor type. (4) Since the header stores the maximum bit depth of the values of the payload, Sensors 1, 2, and 3 store 7($0111_{2}$), 2($0010_{2}$), and 5($0101_{2}$), respectively, and then split-encoded values are stored for each sensor type in the payload.

**Figure 4:**
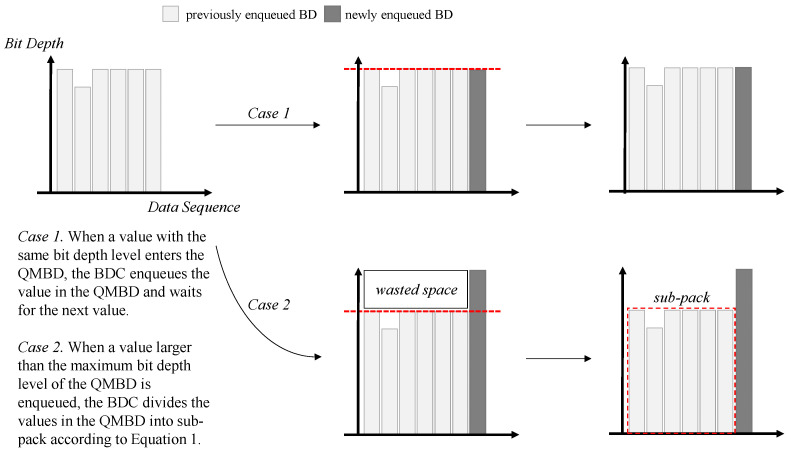
Operations when the same level as the maximum bit depth of QMBD is enqueued (Case 1) and when a value with a bit depth level larger than the maximum bit depth of QMBD is enqueued (Case 2). The red line represents the maximum bit depth of the data stored in the queue.

**Figure 5:**
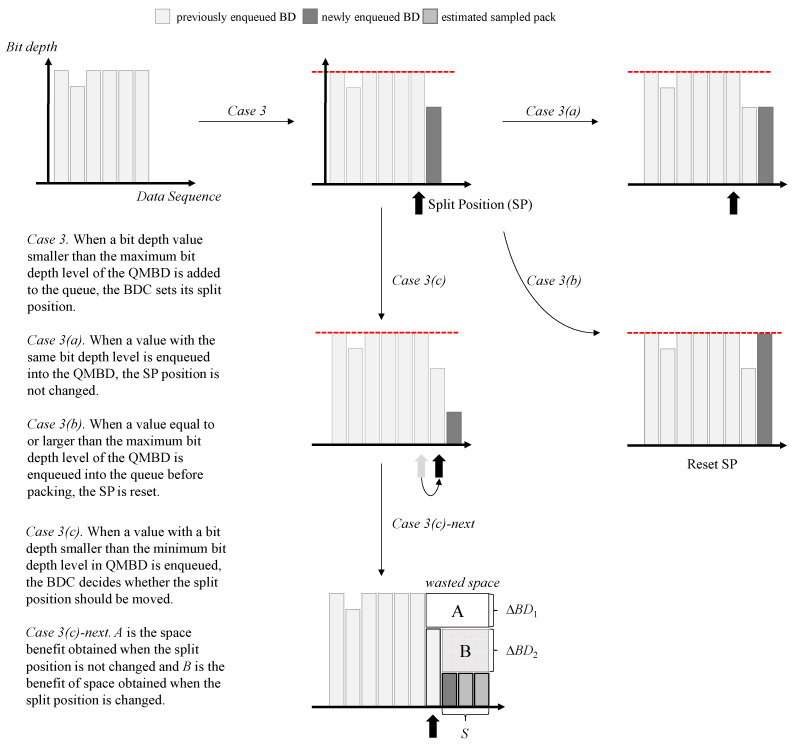
Operations when a bit depth value smaller than the maximum bit depth of the QMBD is enqueued. The red line represents the maximum bit depth of the data stored in the queue.

When we consider Sensor 1 in Figure 3, existing bit packing technology must generate many padded zeros in the numbers stored together because $1010100_{2}$ has a high bit depth level, as seen in the red box of the payload in Sensor 1. When there are many rapidly changing values in the time-series data, such as the values for the Sensor 1 types, the compression efficiency drops sharply. The proposed BDC method aims to reduce the values padded with zeros by dynamically generating multiple sub-packs according to a bit depth. The operating process of the BDC is described in three ways according to the bit depth level of the value enqueuing the QMBD. Figure 4 and Figure 5 show the main operations of the proposed BDC technique in each case.

Figure 4 shows the operation of BDC in two different cases. Case 1 is an operation when a value that is the same level as the maximum bit depth of QMBD is enqueued. Case 2 is an operation when a value having a level larger than the maximum bit depth of QMBD is enqueued. In Figure 4, the horizontal axis indicates the data sequence, and the vertical axis indicates the bit depth level. As in Case 1 in Figure 4, when the bit depth of the value input to QMBD is the same as the maximum bit depth level of QMBD, the value is put into QMBD without any additional operation. On the other hand, as shown in Case 2 in Figure 4, when a value larger than the maximum bit depth of the QMBD is enqueued, the space of the bit pack must be increased by the bit depth level of the currently enqueued value, resulting in space wastage.

Equation (1) is used to calculate the wasted space size. In Equation (1), ${BD}_{new}$ denotes the bit depth level of the currently enqueued new value, maxBD(QMBD) represents the maximum bit depth level in QMBD, and len(QMBD) denotes the length of the QMBD. If the wasted space is larger than the header of the sub-pack, the pack is divided; otherwise, no action is taken.

(1) $$wasted\;space\;size=(BD_{new}-maxBD\left(QMBD\right))\times len\left(QMBD\right)$$

Next, when a bit depth value smaller than the maximum bit depth of the QMBD is inserted into the queue, the BDC sets its split position (SP), as shown in Case 3 in Figure 5. The SP refers to the position that is divided into a sub-pack because the bit depth is too small to be continuously inserted, resulting in wasted bit space. After the SP is set, it can be divided into 3 scenarios again according to the next input bit depth level. First, when a value with the same bit depth level is inserted again into the QMBD, the SP position is unchanged, as shown in Case 3(a) in Figure 5. Second, when a value equal to or larger than the maximum bit depth level of the QMBD is inserted into the queue before packing, the SP is reset, as shown in Case 3(b) in Figure 5. Third, as shown in Case 3(c) in Figure 5, when a value with a bit depth smaller than the minimum bit depth level in the QMBD is enqueued, the BDC decides whether the split position should be moved. As shown in Case 3(c)-next in Figure 5, *A* is the space benefit obtained when the split position is unchanged, and *B* is the benefit of space obtained when the split position is changed. Therefore, the BDC changes the split position when *B* is larger than or equal to *A*; otherwise, it is not changed. The relationship between *A* and *B* can be expressed as Equation (2).

(2) $$\begin{array}{cc} \mathrm{if}\;A\leq B,\;\mathrm{then}\;\mathrm{SP}\;\mathrm{is}\;\mathrm{changed}\\ \;\;\mathrm{else}\;\mathrm{SP}\;\mathrm{is}\;\mathrm{unchanged},\;\mathrm{where}\\ A & =\Delta BD_{1}\times \left(len\left(QMBD_{sp+1}^{n}\right)+S\right)\\ B & =\Delta BD_{2}\times S\\ \Delta BD_{1} & =maxBD\left(QMBD\right)-maxBD\left(QMBD_{sp+1}^{n}\right)\\ \Delta BD_{2} & =maxBD\left(QMBD_{sp+1}^{n}\right)-BD_{n} \end{array}$$

In Equation (2), (sp+1) indicates the position next to the split position, and *n* represents the length of the total entry of the QMDB. $len(QMBD_{sp+1}^{n})$ denotes the length of the QMBD from the position next to the split position to the nth position, $maxBD(QMBD_{sp+1}^{n})$ denotes the maximum bit depth level between the (sp+1)th and nth positions, and *S* denotes the estimated size of the sampled packs. The BDC calculates S using the simple moving average (SMA) or exponential moving average (EMA), as shown in Equation (3).

(3) $$\begin{array}{c} \begin{array}{cc} SMA_{k} & =\frac{1}{k-m+1}\sum_{i=m}^{k}S_{i}\\ EMA_{k} & =\alpha \times S_{k}+\left(1-\alpha \right)\times EMA_{k-1} \end{array} \end{array}$$

In Equation (3), $S_{i}$ is the size of the ith sampled pack, *m* is the number of sampled packs at the start point, and *k* is the number of sampled packs at the end point. Therefore, k−m+1 represents the size of the queue used to monitor the size of the sampled pack. In Equation (3), EMA controls the weighting of $S_{k}$ through parameter α, where we set α to 2/(period+1). The proposed BDC enables real-time compression of sequentially transmitted sensor data by estimating the bit depth size using techniques like SMA or EMA, instead of storing and compressing all the data. This capability makes BDC suitable for compressing sensor data in real-time transmission scenarios.

When the split position is set, the BDC uses Equation (4) to check whether bit pack space is being wasted.

(4) $$\begin{array}{cc} \; & \mathrm{if}\;\;wasted\;space\;size\geq size(sub-header),then\;perform\;bit\;packing\\ \; & \mathrm{else}\;\;go\;to\;next\;step,\;where\;wasted\;space\;size=\\ \; & \;\;\;\;\left(len\left(QMBD\right)-len\left(QMBD_{1}^{sp}\right)\right)\times \left(maxBD\left(QMBD\right)-maxBD\left(QMBD_{sp+1}^{n}\right)\right) \end{array}$$

where $len(QMBD_{1}^{sp})$ denotes the length of the QMBD from the first position to the spth position, and maxBD$\left(QMBD_{sp+1}^{n}\right)$ denotes the maximum bit depth level between the (sp+1)th and nth positions. If the size of the wasted pack space is larger than the size of the sub-header, the BDC divides the $QMBD_{1}^{sp}$ from QMBD, then dequeues the values of $QMBD_{1}^{sp}$ and performs bit packing.

**Figure 6:**
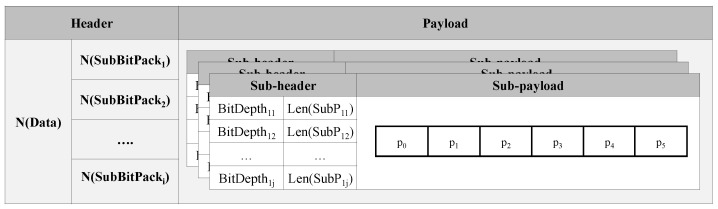
Header and payload structure of bit pack in BDC algorithm.

Figure 6 shows the proposed bit packing frame of the BDC algorithm, and Table 1 shows the description of terms used in Figure 6. The bit packing frame is composed of a header that stores the number of sensor data and the number of sub-bit packs against each datum, and a payload that stores sub-bit packs. Sub-bit packs are composed of sub-headers with the following information: a bit depth level, a sub-payload length, and sub-payloads that store values. In Figure 6, if the total payload length is provided in the header, it is not necessary to include length information for the last sub-payload. However, packet consistency and regularity, we present a uniform packet format, and it is feasible to eliminate the information on the length of the last sub-payload for each sensor, as an implementation perspective.

**Figure 7:**
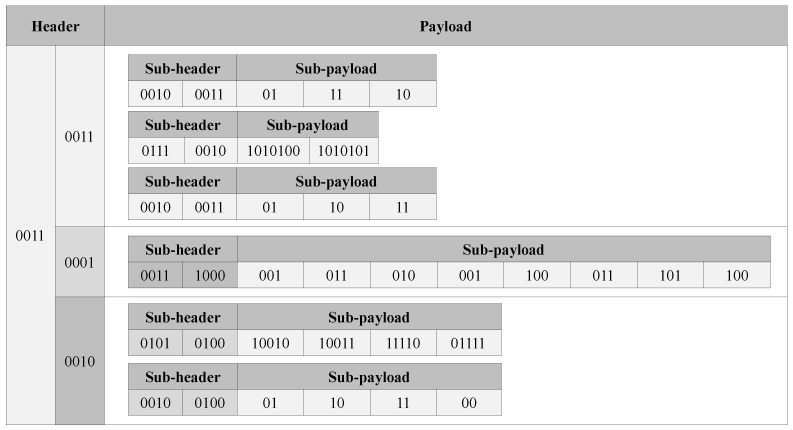
Example of performing the proposed BDC in Figure 3.

Figure 7 shows the result of the proposed BDC technique for Figure 3. The proposed method divides and stores bit packs into several sub-bit packs of different sizes in Sensor 1 and Sensor 3 datasets in Figure 7, because the BDC technique performs compression using dynamic bit packing based on adaptable bit depth level. That is, the data of Sensor 1 is divided into 3 sub-bit packs and each sub-bit pack has 2($0010_{2}$), 7($0111_{2}$) and 2($0010_{2}$) bit depth. In this way, the BDC makes it possible to consume less space compared with representing all data with a 7-bit depth, as shown in Figure 3.

**Figure 8:**
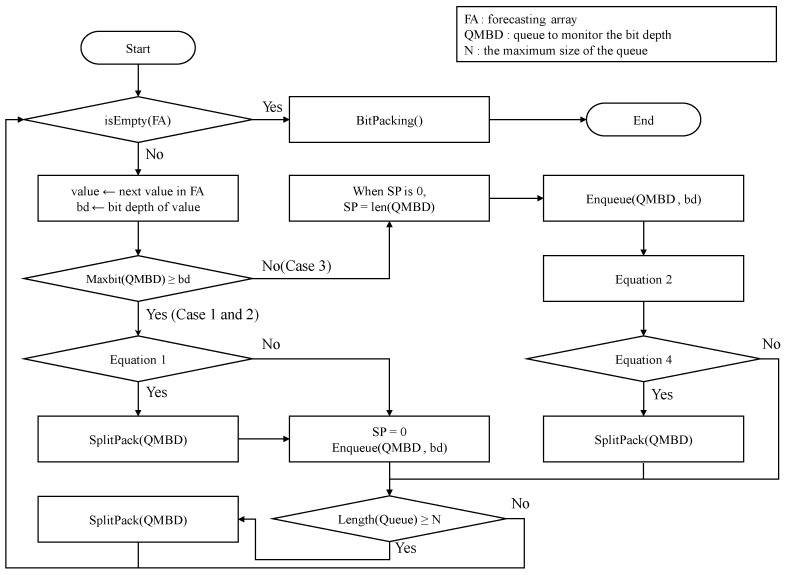
The procedure for the operation of the BDC algorithm.

Finally, Figure 8 shows the overall procedure of the BDC algorithm, including Case 1 to Case 3. As shown in Figure 8, BDC determines the split positions of bit packing based on the space benefit and performs bit packing by dynamically changing its operation according to the bit depth level of the value encoded through the forecasting module.

## 4. Performance Evaluation

In this section, we evaluate the compression efficiency of the proposed BDC method on well-known time-series datasets. We first introduce the datasets used in this study, then show the compression results, and finally discuss the efficiency of our proposals in terms of compression ratio and energy consumption.

### 4.1. Datasets

We used the UCI repository as the main dataset for our performance evaluation. The UCI repository contains various types of data, such as multivariate, univariate, sequential, text, including time-series data, for artificial intelligence and data science. We used the following UCI time-series datasets, and these datasets consist of integers and real numbers. We quantized them to 16-bit integers based on the minimum and maximum values.

Appliances energy prediction dataset: This dataset includes data from the Zigbee wireless sensors used to monitor temperature and humidity at home. The stored data are 10 min averages of sensor data collected over a period of 4.5 months [36].

Air quality dataset: This dataset contains air quality data collected on Italian roads from March 2004 to February 2005. The dataset includes CO, non-metallic hydro-carbons, benzene, total nitrogen oxides (NOx), and nitrogen dioxide (NO${}_{2}$) levels with missing values set to −200 [37].

Gas sensor array temperature modulation dataset: This dataset contains measurements from chemical sensors exposed to gas mixtures. It includes CO concentration, temperature, humidity, flow rate, and data from 14 MOx gas sensors inside a gas chamber. The results obtained on 30 September 2016 were used in this study [38].

SML2010 dataset: This dataset is a collection of time-series data collected from a smart home. The dataset includes data on indoor and outdoor temperature, humidity, illumination, and other variables, collected over approximately 28 days [39].

### 4.2. Experimental Results and Discussion

We compared the compression ratio and energy consumption of the BDC method with those of representative bit packing algorithms such as Sprintz with XOR or delta encoding applied as forecasting, and TSXor [40]. The raw data were stored by performing bit packing based on the largest bit depth among the error values obtained from the FM. The length of the QMBD was set to 16, and this size was enough to monitor time-series data with several multidimensional variations in our study.

**Figure 9:**
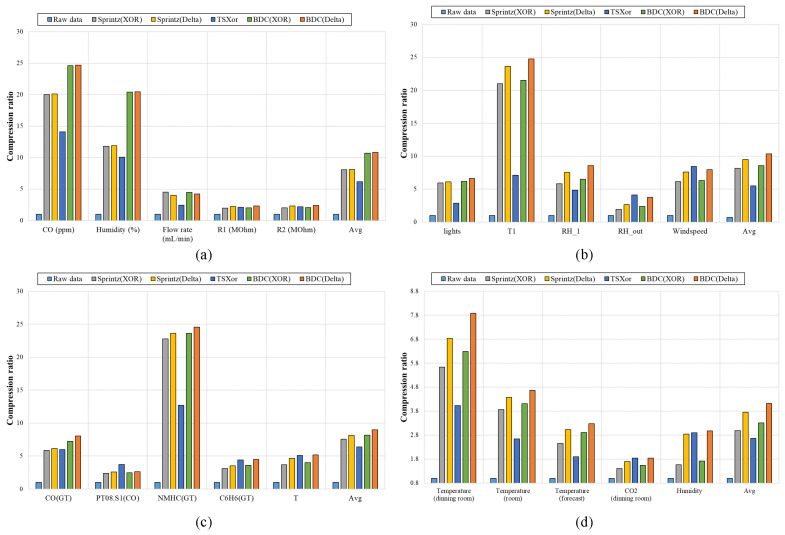
Compression ratio of each algorithm according to the dataset: (**a**) Gas sensor array temperature modulation dataset, (**b**) Appliances energy prediction dataset, (**c**) Air Quality dataset, and (**d**) SML2010 dataset.

Figure 9 shows the compression ratio of each algorithm based on four datasets. The compression ratio is defined as the ratio between the size of the original data (Original Size) and the size of the compressed data (Compressed Size). It can be expressed as Compression ratio=Original Size/Compressed Size. In the graph, the x-axis represents the type of sensor data, and the y-axis represents the compression ratio. In Figure 9, the XOR and delta algorithms are the encoding techniques that were chosen to be applied as prediction algorithms, which are represented in combination with bit packing algorithms Sprintz and BDC, respectively. As shown in Figure 9, the BDC method shows a high compression ratio for most datasets. Among the forecasting algorithms, delta encoding exhibits a higher compression ratio than XOR encoding. The combined delta forecasting and BDC algorithm has a compression ratio up to 24.7 times higher than that of the raw data on the datasets used in the experiment. Compared with Sprintz’s bit packing, the BDC algorithm improves the compression ratio by up to 73%, and 14% on average, when using XOR forecasting and improves compression ratio by up to 72%, and 15% on average, when using delta forecasting. Furthermore, compared with the TSXor compression algorithm, the proposed BDC algorithm shows a maximum performance improvement of 247% and an average improvement of 64%. The proposed BDC algorithm has a relatively high compression ratio, resulting in a reduced space requirement for the buffer before data transmission as well as a decrease in the payload of transmitted packets. In the experiments conducted, the reduction rate of the payload was, on average, 28% for XOR forecasting and 32% for delta forecasting. In comparison with TSXor, the proposed method demonstrated a maximum payload reduction of 98% and an average reduction of 46%.

**Figure 10:**
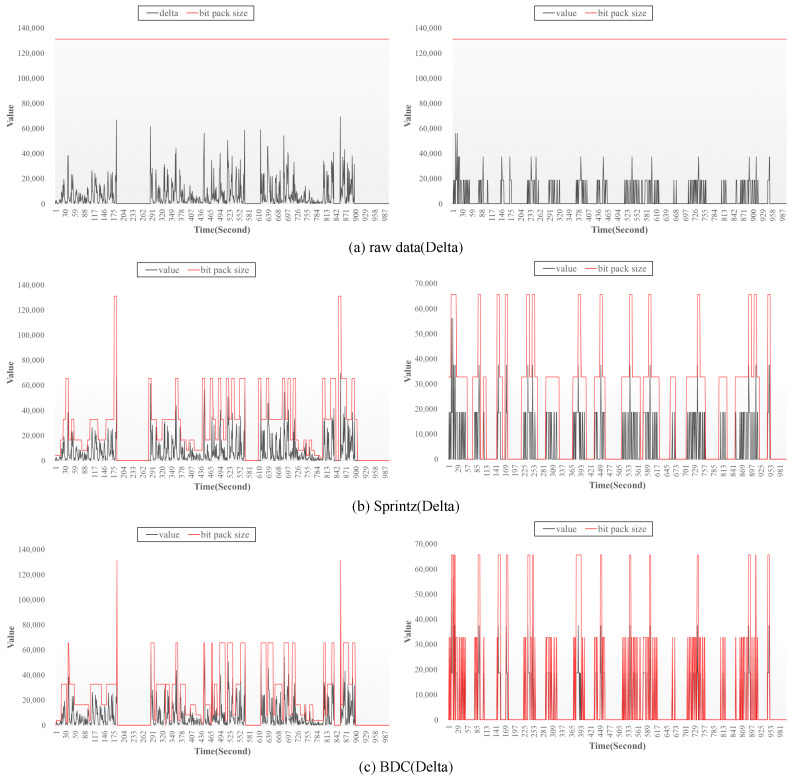
A size comparison of bit packing according to the operation of compression algorithms: (**a**) raw data, (**b**) Sprintz, (**c**) BDC. In each graph (**a**–**c**), the left is Air auality dataset and the right is Appliances energy prediction dataset, respectively.

The performance enhancement of our proposed method shown in Figure 9 mainly originates from dynamic bit packing by adaptively changing the packing size, and Figure 10 shows the actual compression process and its analysis. Figure 10 shows the results of the size comparison of bit packing according to the operation of (a) raw data (delta) and the bit pack performed by the (b) Sprintz (delta) algorithms and the proposed (c) BDC (delta) algorithm. In Figure 10, the left and right figures are NMHC (GT) in the Air quality dataset and lights in the Appliance energy prediction dataset, respectively. In each graph, the x-axis represents the sequence of time series data, and the y-axis represents the encoded values after applying the delta encoding and zigzag algorithm. The black line in the graph is the data value extracted via delta encoding, and the red line is the size of the bit packing. Because the bit packing size of the raw data is acheived by performing compression on the entire dataset, a large amount of space is wasted due to some data with high bit depth. Compared with raw data, Sprintz and BDC achieve higher compression efficiency because they perform bit packing based on sampled data rather than the entire dataset. Compared with the Sprintz algorithm, however, which makes use of a fixed-size sampling procedure, the BDC algorithm is able to dynamically control the sampling size, thereby increasing the bit packing efficiency for data with high variance. Through dynamic bit packing, the BDC separates outlier values using sub-bit packing and includes values with similar bit depth in the same sampling to reduce wasted space.

A comparison of the energy consumption of our proposed method with that of other algorithms is as follows: For comparing the energy consumption, we used a TI SensorTag C2650 [41], and the detailed specifications of this terminal device are shown in Table 2. The energy consumption of the sensor device is composed of the energy needed for the computation of the compression process, the energy used for writing the compressed data to memory, and the energy required for transmission through Bluetooth. The following shows the energy consumption for each part.

First, the computational energy of the compression process is the sum of the energy components proportional to the number of clocks in the microcontroller for data processing, including the standby power and typical active power. When activated with an average clock of 24 MHz (average) and the basic power of 61 µA (shown in Table 2), the average load current expected is 24 MHz × (6.1 × $10^{-5})$ µA/MHz = 1.46 mA. Therefore, assuming that the operating voltage (indicated by $V_{DDS}$) of a typical MCU is 3.0 V, the energy consumption of the MCU is expected to be (1.46 × $10^{-3})$ A × 3 V =4.38 × $10^{-3}$ W.

Second, the energy used for writing to memory is as follows: The C2650 device consisted of 128 KB of flash memory area to store code and data and 4 KB + 2 × 6 KB of SRAM space. The proposed system used the entire 128 KB flash memory space for data storage. Table 3 shows the energy consumption characteristics according to operations in the C2650 device. Because the total memory capacity of the terminal device was 128 KB and the page size was 4 KB, 32 page slots were allocated. For each 4-byte write, 8.15 mA of energy was consumed for a period of 8 µs. Therefore, 3.0 V × (8.15 × $10^{-3}$ A × (8 × $10^{-6}$) s × $10^{3})$ = 187.5 × $10^{-6}$ W ($V_{DDS}$ = 3.0 V) of energy was consumed for writing to one page (4 KB) of memory, and thus 6 × $10^{-3}$ W of energy was consumed for writing to the entire space (128 K). Since an erasure in NAND flash is applied to the entire memory, 3 V × (12.6 × $10^{-3}$) A × (8 × $10^{-3}$) × 32 ms = 9.679 × $10^{-3}$ W of energy was required. Therefore, in the C2650 device, 6 × $10^{-3}$ W (which is writing entire memory) + 9.679 × $10^{-3}$ W (which is erasing entire memory before writing) = 15.679 × $10^{-3}$ W of energy was consumed per 128 KB of data size, respectively.

Finally, the energy required for transmission was as follows: The maximum Bluetooth data payload per packet was 251 bytes. Table 4 lists the Bluetooth power consumption parameters based on an input voltage of $V_{DDS}$ 3 V, output power of 0 ddBM, advertising interval of 1000 ms, connection interval of 1000 ms, and payload data size of 251 bytes. Based on Table 4, the average current draw during a connection event $I_{e}$ is 4325.247 µs × mA/850.095 µs = 5088.6 µA. The average current for the entire connection interval can be calculated by Equation (5) [42].

(5) $$\begin{array}{c} \begin{array}{cc} \;I_{average} & =\frac{\left(t_{interval}-t_{awake})\times I_{standby}+(t_{awake}\times I_{event}\right)}{t_{interval}} \end{array} \end{array}$$

According to Equation (5), the average current draw during connection is calculated as (100 ms − 5.101 ms) × 1 µA + (5.101 ms × 5088.6 µA)/100 ms = 260.5 µA. Thus, the energy consumed per packet is 3 V × 2.605 × $10^{-4}$ A = 7.815 × $10^{-4}$ W. Since each packet can transmit tahe maximum of 251 bytes of data, it would take 510 transmission operations to transmit all 128 KB of data. Therefore, the total energy consumption would be 3.986 × $10^{-1}$ W, obtained by multiplying the energy consumed per packet by the number of packets required (510). The aforementioned calculation assumes that the device is connected as a peripheral for data transmission, and it does not take into account any fluctuations in power consumption resulting from the use of other sensors. Therefore, the actual energy consumption can vary depending on other external factors such as transmission power, transmission duration, and Bluetooth module efficiency.

Based on this analysis, the total energy consumption can be determined using Equation (6), for operation duration *t* (second) and data size *d* (byte).

(6) $$\begin{array}{c} \begin{array}{cc} \mathrm{Total}\;\mathrm{energy}\;\mathrm{consumption}= & \mathrm{compression}\;\mathrm{energy}\\ \; & +\;\mathrm{write}\;\mathrm{energy}\\ \; & +\;\mathrm{transmission}\;\mathrm{energy}\\ = & (t\times \left(4.38\times 10^{-3}\right))W\\ \; & +(d/128\;\mathrm{KB}\times \left(15.679\times 10^{-3}\right))W\\ \; & +(d/128\;\mathrm{KB}\times \left(3.986\times 10^{-1}\right))W \end{array} \end{array}$$

**Figure 11:**
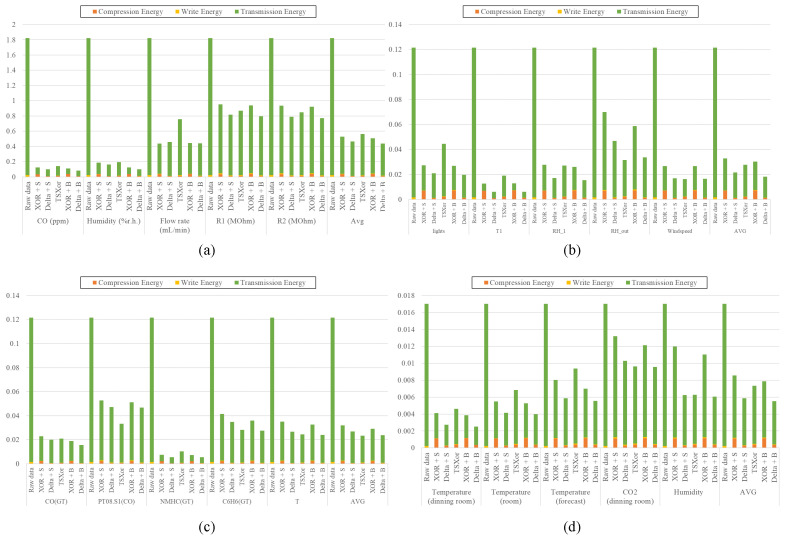
Energy consumption of the proposed BDC and other algorithms: (**a**) Gas sensor array temperature modulation dataset, (**b**) Appliances energy prediction dataset, (**c**) Air quality dataset, and (**d**) SML2010 dataset.

Figure 11 shows the energy consumption for the raw data and each compression method by applying Equation (6). In Figure 11, the x-axis represents the types of sensor data, and the y-axis shows energy consumption. In Figure 11, the graph illustrates different aspects of energy consumption using different colors. When compared with other compression techniques, the BDC algorithm demonstrates slightly higher computational complexity due to the calculations involved in BDE. However, the BDC algorithm achieves a higher compression ratio, resulting in reduced numbers of compressed packets and transmission time, consequently leading to lower write and transmission energies. As shown in Figure 11, the Sprintz algorithm increases energy consumption via the compression of MCU for some data. TSXor also consumes relatively more energy compared with the proposed method due to its lower compression ratio, making it less efficient for data transmission. The BDC algorithm also increases the energy consumption owing to the MCU calculation, but the higher data compression rate reduces the energy consumption of data transmission. In sensor devices, the energy consumption of data transmission often outweighs that of computation due to the higher power requirements of wireless communication modules, which have to operate over varying distances or potentially through different environmental conditions. Therefore, minimizing the amount of data to be transmitted becomes a critical factor in reducing overall energy consumption, and the BDC effectively reflects these advantages. As demonstrated in Figure 11, the BDC resulted in an energy consumption reduction of up to 94% and 38%, with an average reduction of 69% and 12% compared with that of the raw data and Sprintz, respectively. Furthermore, compared with TSXor, the proposed method reduced energy consumption by up to 67%, with an average of 24%.

## 5. Conclusions

With technologies such as smart factories, smart farms, autonomous vehicles, and digital twins becoming increasingly pervasive across various industries, the amount of data generated by sensors has also increased. These data can be transmitted to edge computers and cloud servers for data analysis or model generation in the field of artificial intelligence or data science.

In this study, we developed a BDC technique to compress the time-series data generated by sensors. The BDC algorithm performs bit packing by dynamically determining the pack size based on the pattern of the bit depth level of sensor data, thereby maximally reducing the space wastage that may occur during the bit packing process. The BDC technique compresses time-series data transmitted from an end device to an edge device or from an edge device to a cloud data center. Because the BDC is a lossless compression technique, there is no loss of information in the collected sensor data, which is especially useful as training data in the field of artificial intelligence or predictive analysis of data science. The lossless feature of the BDC can more accurately reflect outliers or variations, that is, the data robustness. In the experiment, the BDC demonstrated an improvement in the compression ratio of up to 247% and 30% on average, and a reduction in power consumption of up to 34%, with an average of 18%, compared with the other algorithms. This implies that the proposed BDC has high utility value for compressing sensor data transmitted from a wireless terminal device to an edge computer or a cloud data center.

As future research, we plan to study an enhanced forecasting model to further improve the compression ratio of time-series data. In addition, we are planning a study on the reduction in computation overhead in edge nodes for lossless compression.

## Figures and Tables

**Table 1:** Description of terms in Figure 6.

Symbol	Description
N(Data)	The number of sensor data
N(${SubBitPack}_{i}$)	The number of sub-bit packs of ith data in header
${BitDepth}_{ij}$	The bit depth of jth sub bit pack in ith data
Len(${SubP}_{ij}$)	The payload length of jth sub bit pack in ith data

**Table 2:** Energy consumption parameters of TI SensorTag C2650.

Test Conditions	Typical	Unit
Core Frequency	Up to 48	MHz
Operation Voltage	1.8 to 3.8	C
Input Voltage ($V_{DDS}$)	3.0	V
Standby Current	1	µA
Shutdown Current	100	nA
Active-Mode RX	5.9	mA
Active-Mode TX at 0 dBm	6.1	mA
Active-Mode TX at +5 dBm	9.1	mA
Active-Mode MCU	61	µA/MHz
Active-Mode Sensor Controller	8.2	µA/MHz

**Table 3:** Flash memory characteristics of TI SensorTag C2650.

Parameter	Test Conditions	Typical	Unit
Input voltage, respectively ($V_{DDS}$)	-	3.0	V
In-system programmable flash size	-	128	KB
Flash page/sector size	-	4	KB
Flash page/sector erase current	Average delta current	12.6	mA
Flash write current	Average delta current, 4 bytes at a time	8.15	mA
Flash page/sector erase time	-	8	ms
Flash write time	4 bytes at a time	8	µs

**Table 4:** The parameters of energy consumption for Bluetooth low-energy controller, IEEE 802.15.4 in SensorTag C2650.

State	Time [µs]	Current [mA]	Time × Current
Wake Up and Pre-processing	1283.89	3.10	3981.68
Preparation for Receive	394.22	3.58	1409.55
Receive (RX)	461.33	6.69	3085.90
RX to TX transition	109.22	5.21	568.97
Transmit (TX)	1998.47	7.34	14,669.14
Post-Processing	853.44	2.62	2239.63
Average Value of Connection	850.095	4.7567	4325.247
Total Value of Connection	5100.57	28.54	25954.9

## Data Availability

Not applicable.
